# Supplementary material for: RANTES and CD40L under Conditions of Long-Term Physical Exercise: A Potential Link to Adaptive Immunity
Source: Int J Environ Res Public Health. 2022 Jul 16;19(14):8658. doi: 10.3390/ijerph19148658 (PMC9316936; doi:10.3390/ijerph19148658)
Supplement: Supplementary file 1 [file ijerph-19-08658-s001.zip › ijerph-1752897-supplementary.pdf]

Supplementary Materials:

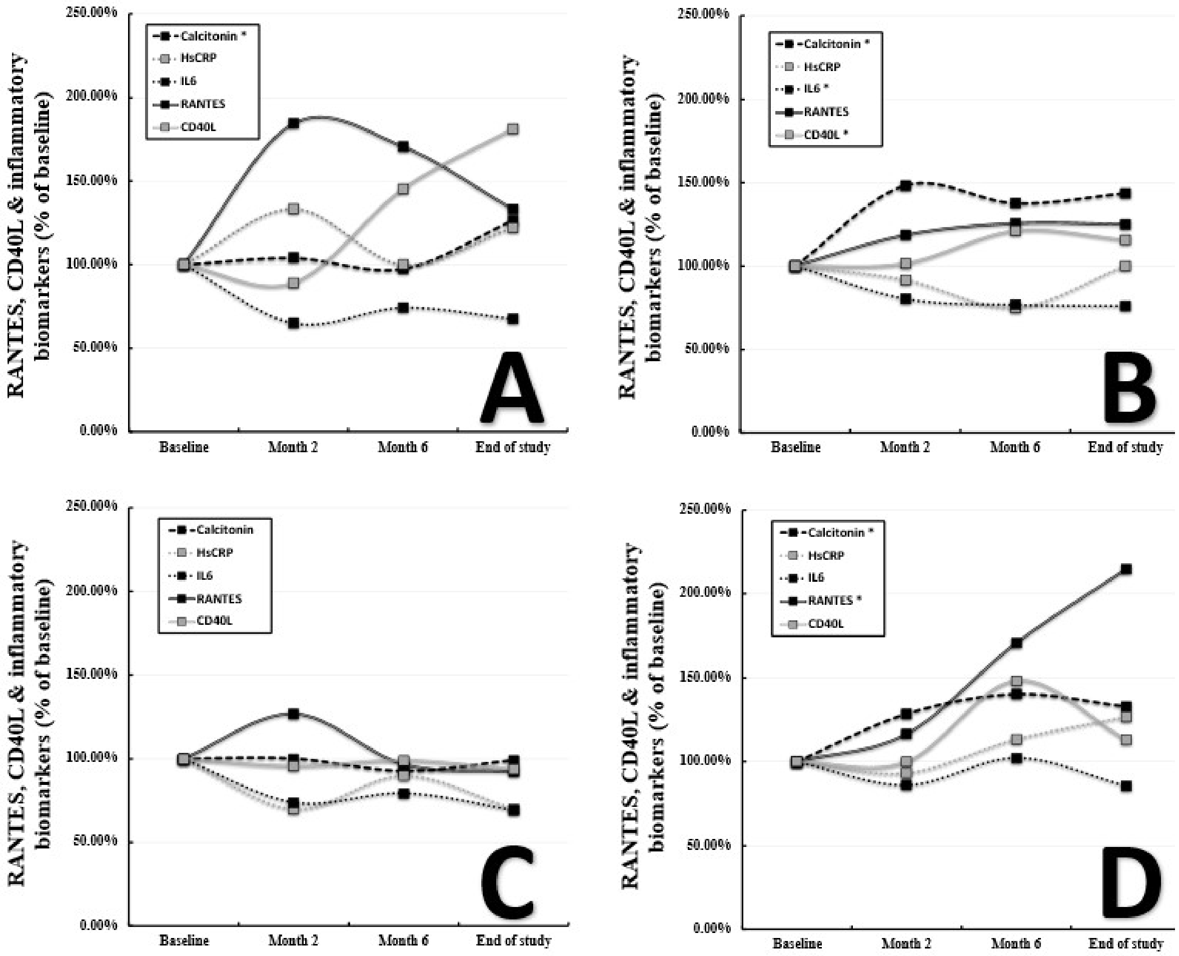

Supplementary Figure S1. Progression of RANTES, CD40L, hsCRP, IL6, and calcitonin serum levels in subgroups **α** (initial performance < 100%, performance gain ≤ 2.9%, n=9, Supplementary Figure 1A), **β** (initial performance < 100%, performance gain > 2.9%, n=32, Supplementary Figure 1B), **γ** (initial performance ≥ 100%, performance gain ≤ 2.9%, n=18, Supplementary Figure 1C), and **δ** (initial performance ≥ 100%, performance gain > 2.9%, n=39, Supplementary Figure 1D) over the course of 8 months. ”*” indicates a two-tailed p-value of $p < 0.05$ comparing baseline values to those at the end of the study.

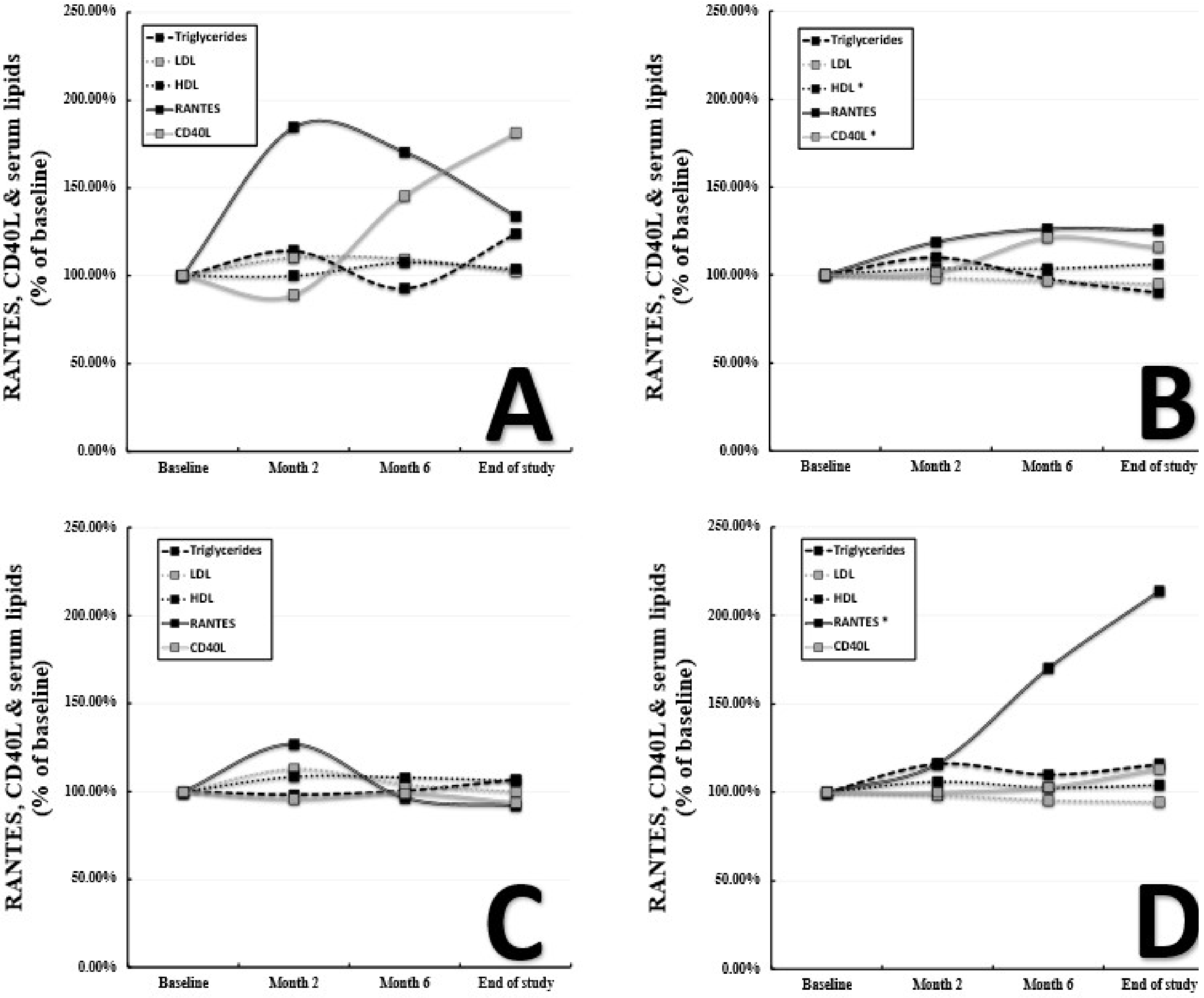

Supplementary Figure S2. Progression of RANTES, CD40L, HDL, LDL, and serum triglycerides in subgroups **α** (initial performance < 100%, performance gain ≤ 2.9%, n=9, Supplementary Figure 2A), **β** (initial performance < 100%, performance gain > 2.9%, n=32, Supplementary Figure 2B), **γ** (initial performance ≥ 100%, performance gain ≤ 2.9%, n=18, Supplementary Figure 2C), and **δ** (initial performance ≥ 100%, performance gain > 2.9%, n=39, Supplementary Figure 2D) over the course of 8 months. ”*” indicates a two-tailed p-value of $p < 0.05$ comparing baseline values to those at the end of the study.

**RANTES** Risk factors:	Group α Gain ≤2.9% (n=9)	Group β Gain> 2.9% (n=32)	Group γ Gain ≤2.9% (n=18)	Group δ Gain> 2.9% (n=39)	Total cohort (n=98)
Packyears	0.397	0.262	0.965	0.569	0.851
Diabetes mellitus	0.272	0.786	0.644	0.630	0.326
Hypertension	0.638	0.355	0.113	0.107	0.775
Dyslipidemia	0.815	0.535	0.633	0.255	0.327
Overweight	0.946	0.822	0.042 (-0.484)*	0.378	0.555
Positive family history	0.638	0.444	0.833	0.231	0.697
**sCD40** Risk factors:	Group α Gain ≤2.9% (n=9)	Group β Gain> 2.9% (n=32)	Group γ Gain ≤2.9% (n=18)	Group δ Gain> 2.9% (n=39)	Total cohort (n=98)
Packyears	0.388	0.875	0.414	0.277	0.226
Diabetes mellitus	0.635	0.094	0.303	0.289	0.185
Hypertension	0.730	0.067	0.654	0.248	0.083
Dyslipidemia	0.323	0.494	0.829	0.120	0.255
Overweight	0.901	0.609	0.179	0.669	0.977
Positive family history	0.051	0.527	0.524	0.810	0.296

Supplementary Table S1. RANTES and CD40L and pre-existing risk factors at baseline. ”*” indicates a two-tailed p-value of $p < 0.05$.

**RANTES** Parameters:	**Group α baseline** Gain ≤2.9% (n=9)	**Group β baseline** Gain> 2.9% (n=32)	**Group γ baseline** Gain ≤2.9% (n=18)	**Group δ baseline** Gain> 2.9% (n=39)
Calcitonin	0.036 (+0.700)*	0.421	0.824	0.151 (+0.275)
IL6	0.013 (-0.783)*	0.155	0.651	0.090 (+0.275)°
hsCRP	0.008 (-0.814)*	0.067 (+0.328)°	0.996	0.668
**RANTES** Parameters:	**Group α end of study** Gain ≤2.9% (n=9)	**Group β end of study** Gain> 2.9% (n=32)	**Group γ end of study** Gain ≤2.9% (n=18)	**Group δ end of study** Gain> 2.9% (n=39)
Calcitonin	0.221 (-0.006)	0.973	0.441	0.014 (+0.392)*
IL6	0.347 (-0.087)	0.634	0.596	0.232 (-0.196)
hsCRP	0.559 (+0.108)	0.556 (+0.108)	0.577	0.470
**sCD40** Parameters:	**Group α baseline** Gain ≤2.9% (n=9)	**Group β baseline** Gain> 2.9% (n=32)	**Group γ baseline** Gain ≤2.9% (n=18)	**Group δ baseline** Gain> 2.9% (n=39)
Calcitonin	0.551	0.936	0.272	0.151 (+0.235)
IL6	0.205	0.482	0.225	0.090 (+0.275)°
hsCRP	0.695	0.060 (+0.336)°	0.993 (-0.022)	0.668
**sCD40** Parameters:	**Group α end of study** Gain ≤2.9% (n=9)	**Group β end of study** Gain> 2.9% (n=32)	**Group γ end of study** Gain ≤2.9% (n=18)	**Group δ end of study** Gain> 2.9% (n=39)
Calcitonin	0.660	0.168	0.154	0.015 (+0.386)*
IL6	0.179	0.705	0.121	0.217 (-0.196)
hsCRP	0.342	0.224 (+0.221)	0.019 (+0.545)*	0.691

Supplementary Table S2. Correlations of RANTES and CD40L with hsCRP, IL6, and calcitonin serum levels throughout the study period. ”*” indicates a two-tailed p-value of $p < 0.05$. “°” indicates a borderline significance of $p = 0.05$, $p = 0.1$.

Parameters:	Group α baseline Gain ≤2.9% (n=9)	Group β baseline Gain> 2.9% (n=32)	Group γ baseline Gain ≤2.9% (n=18)	Group δ baseline Gain> 2.9% (n=39)
Triglycerides	0.898	0.766	0.743	0.888
HDL	0.651	0.364	0.284	0.468
LDL	0.050 (-0.667)°	0.615	0.395	0.013 (+0.393)*
**RANTES** Parameters:	Group α end of study Gain ≤2.9% (n=9)	Group β end of study Gain> 2.9% (n=32)	Group γ end of study Gain ≤2.9% (n=18)	Group δ end of study Gain> 2.9% (n=39)
Triglycerides	0.898	0.884	0.261	0.486
HDL	0.589	0.882	0.645	0.199
LDL	0.460 (+0.283)	0.308	0.416	0.875 (+0.026)
**CD40L** Parameters:	Group α baseline Gain ≤2.9% (n=9)	Group β baseline Gain> 2.9% (n=32)	Group γ baseline Gain ≤2.9% (n=18)	Group δ baseline Gain> 2.9% (n=39)
Triglycerides	0.575	0.349	0.768	0.521
HDL	0.814	0.503	0.269	0.226
LDL	0.546	0.028 (+0.389)*	0.456 (+0.194)	0.078 (+0.286)°
**CD40L** Parameters:	Group α end of study Gain ≤2.9% (n=9)	Group β end of study Gain> 2.9% (n=32)	Group γ end of study Gain ≤2.9% (n=18)	Group δ end of study Gain> 2.9% (n=39)
Triglycerides	0.154	0.626	0.510	0.165
HDL	0.284	0.894	0.714	0.270
LDL	0.406	0.077 (+0.317)°	0.037 (+0.494)*	0.472 (+0.119)

Supplementary Table S3. Correlations of RANTES and CD40L with HDL, LDL, and serum triglycerides throughout the study period. ”*” indicates a two-tailed p-value of $p < 0.05$. “°” indicates a borderline significance of p = 0.05, p = 0.1.

Table 1	**Group α**	**Group β**	**Group γ**	**Group δ**	**Total cohort**
**Parameters:**	**Gain ≤2.9% (n=9)**	**Gain> 2.9% (n=32)**	**Gain ≤2.9% (n=18)**	**Gain> 2.9% (n=39)**	**(n=98)**
**RANTES 0 (ng/mL)**	**1.65 ± 5.02**	**2.14 ± 1.61**	**2.60 ± 4.05**	**1.88 ± 1.37**	**2.08 ± 2.13**
**RANTES 2 (ng/mL)**	**3.05 ± 1.10**	**2.54 ± 1.97**	**3.29 ± 2.88**	**2.19 ± 1.44**	**2.59 ± 1.95**
**RANTES 6 (ng/mL)**	**2.82 ± 1.42**	**2.70 ± 1.57**	**2.50 ± 1.80**	**3.21 ± 2.64**	**2.87 ± 2.06**
**RANTES 8 (ng/mL)**	**2.21 ± 0.70**	**2.69 ± 1.76**	**2.39 ± 1.59**	**4.03 ± 3.12**	**3.12 ± 2.42**
**CD40L 0 (pg/mL)**	**320.83 ± 234.02**	**323.10 ± 585.66**	**213.49 ± 100.38**	**199.25 ± 75.73**	**253.47 ± 348.78**
**CD40L 2 (pg/mL)**	**285.82 ± 263.52**	**328. 48 ± 561.47**	**204.75 ± 82.96**	**199.38 ± 76.94**	**250.18 ± 335.38**
**CD40L 6 (pg/mL)**	**465.83 ± 553.75**	**392.96 ± 912.44**	**211.93 ± 108.78**	**205.67 ± 54.51**	**293.08 ± 548.11**
**CD40L 8 (pg/mL)**	**580.89 ± 1155.99**	**374.19 ± 615.96**	**200.32 ± 65.54**	**225.11 ± 79.13**	**301.91 ± 497.90**
**HsCRP 0 (mg/dL)**	**0.09 ± 0.04**	**0.23 ± 0.20**	**0.20 ± 0.30**	**0.14 ± 0.15**	**0.18 ± 0.20**
**HsCRP 2 (mg/dL)**	**0.11 ± 0.06**	**0.21 ± 0.22**	**0.13 ± 0.10**	**0.13 ± 0.17**	**0.16 ± 0.17**
**HsCRP 6 (mg/dL)**	**0.08 ± 0.04**	**0.17 ± 0.13**	**0.17 ± 0.20**	**0.37 ± 1.24**	**0.24 ± 0.78**
**HSCRP 8 (mg/dL)**	**0.10 ± 0.06**	**0.24 ± 0.29**	**0.14 ± 0.12**	**0.19 ± 0.53**	**0.19 ± 0.38**
**Calcitonin 0 (pg/mL)**	**1.24 ± 1.35**	**2.14 ± 3.10**	**3.01 ± 3.41**	**2.30 ± 1.83**	**2.27 ± 2.59**
**Calcitonin 2 (pg/mL)**	**1.28 ± 1.49**	**3.18 ± 3.92**	**3.00 ± 2.93**	**2.97 ± 2.22**	**2.88 ± 2.97**
**Calcitonin 6 (pg/mL)**	**1.21 ± 1.29**	**2.96 ± 3.31**	**2.79 ± 2.98**	**3.23 ± 2.72**	**2.86 ± 2.88**
**Calcitonin 8 (pg/mL)**	**1.56 ± 1.71**	**3.09 ± 3.82**	**2.98 ± 2.82**	**3.06 ± 2.29**	**2.92 ± 2.93**
**IL6 0 (pg/mL)**	**2.71 ± 1.22**	**3.06 ± 2.16**	**2.48 ± 2.41**	**2.23 ± 0.85**	**2.59 ± 1.74**
**IL6 2 (pg/mL)**	**1.76 ± 1.17**	**2.46 ± 1.78**	**1.82 ± 1.38**	**1.93 ± 1.31**	**2.07 ± 1.49**
**IL6 6 (pg/mL)**	**2.01 ± 1.47**	**2.35 ± 1.40**	**1.96 ± 1.31**	**2.29 ± 1.85**	**2.22 ± 1.57**
**IL6 8 (pg/mL)**	**1.84 ± 1.49**	**2.33 ± 1.57**	**1.72 ± 1.12**	**1.92 ± 1.96**	**2.01 ± 1.66**
**Triglycerides 0 (mg/dL)**	**154.00 ± 85.85**	**149.09 ± 99.70**	**110.52 ± 72.02**	**119.30 ± 62.20**	**130.81 ± 80.67**
**Triglycerides 2 (mg/dL)**	**176.33 ± 118.08**	**164.93 ± 98.09**	**108.38 ± 28.07**	**138.63 ± 80.80**	**144.98 ± 85.96**
**Triglycerides 6 (mg/dL)**	**143.00 ± 54.42**	**146.21 ± 81.08**	**110.88 ± 79.07**	**131.27 ± 77.14**	**133.24 ± 76.71**
**Triglycerides 8 (mg/dL)**	**191.55 ± 87.74**	**134.25 ± 70.68**	**118.00 ± 70.76**	**138.28 ± 80.73**	**138.13 ± 77.50**
**LDL 0 (mg/dL)**	**126.20 ± 50.35**	**117.02 ± 32.24**	**111.65 ± 28.67**	**116.26 ± 34.96**	**116.62 ± 34.36**
**LDL 2 (mg/dL)**	**139.43 ± 48.48**	**115.85 ± 35.60**	**125.98 ± 40.46**	**114.43 ± 28.40**	**119.40 ± 35.57**
**LDL 6 (mg/dL)**	**138.40 ± 56.19**	**113.43 ± 32.06**	**116.29 ± 33.36**	**111.06 ± 33.15**	**115.54 ± 35.10**
**LDL 8 (mg/dL)**	**129.35 ± 43.28**	**111.21 ± 30.95**	**111.62 ± 25.165**	**110.06 ± 33.152**	**112.49 ± 32.12**
**HDL 0 (mg/dL)**	**52.22 ± 19.46**	**56.09 ± 21.65**	**61.94 ± 11.97**	**60.46 ± 14.99**	**58.51 ± 17.47**
**HDL 2 (mg/dL)**	**52.66 ± 20.60**	**58. 06 ± 23.01**	**67.94 ± 11.97**	**64.07 ± 17.81**	**61.65 ± 19.32**
**HDL 6 (mg/dL)**	**56.667 ± 19.83**	**58.35 ± 19.83**	**66.94 ± 12.82**	**62.05 ± 15.37**	**61.28 ± 16.98**
**HDL 8 (mg/dL)**	**54.88 ± 20.42**	**59.46 ± 19.57**	**65.77 ± 13.45**	**62.92 ± 17.09**	**61.58 ± 17.68**

Supplementary Table S4. Mean ± standard deviation of the analyzed parameters throughout the study period. HsCRP, high-sensitivity C-reactive protein; IL6, interleukin 6; LDL, low-density lipoprotein; HDL, high-density lipoprotein.
